# Analysis of the Iranian maternal mortality surveillance system and providing system improvement strategies: study protocol for strategy formulation

**DOI:** 10.1186/s12978-020-00963-2

**Published:** 2020-07-23

**Authors:** Marjan Beigi, Shahideh Jahanian Sadatmahaleh, Nasrin Changizi, Eesa Mohammadi, Ashraf Kazemi

**Affiliations:** 1 Department of Midwifery and Reproductive Health, Faculty of Medical Sciences, Tarbiat Modares University, Tehran, Iran; 2 Ministry of Health and Medical education, Tehran, Iran; 3 Department of Nursing, Faculty of Medical Sciences, Tarbiat Modares University, Tehran, Iran; 4 Nursing and Midwifery Care Research Center, Department of Midwifery and Reproductive Health, Faculty of Nursing and Midwifery, Isfahan University of Medical Sciences, Isfahan, Iran

**Keywords:** Mortalité maternelle, Etude de la mortalité maternelle, Système de surveillance de la mortalité des mères iraniennes, Surveillance et réponse de la mortalité maternelle, Stratégie, Maternal mortality, Maternal death review, Iranian maternal mortality surveillance system, Maternal death surveillance and response, Strategy

## Abstract

**Background:**

The implementation of the maternal mortality surveillance system in Iran has significantly reduced the incidence of maternal mortality. However, the pattern of the causes of the mortalities, which has remained constant over the years, are still concerning. This study aimed to explain the experiences of the actors of the Iranian maternal mortality surveillance and provide strategies for improving this system.

**Methods:**

This research is a qualitative study to develop strategies, that will be conducted in two phases. In the first phase, purposive sampling will be performed, and the data will be collected based on the experiences of the Iranian maternal mortality surveillance system actors in Iran’s Ministry of Health and the selected universities (Shiraz, Isfahan, Tehran, Zahedan, Alborz, Shahrekord) through semi-structured interviews. Moreover, during this phase, some part of the data will be collected through random participation of the researcher in some maternal mortality committees of the selected universities. In order to carry out the second phase, a panel of experts will be set up to discuss the best strategies for improving the Iranian maternal mortality surveillance by considering the above results.

**Discussion:**

The analysis of maternal mortality surveillance system needs to evaluate the experiences of the actors who are the policymakers of this system and can be effective in identifying its challenges. This analysis and formulation of the subsequent strategies can lead to maternal health indicators remaining within the range of international standards or even beyond those standards in Iranian universities and countries with similar surveillance system.

## Résumé en français (French abstract)

**Contexte de recherché:**

La mise en œuvre du système de surveillance de la mortalité maternelle en Iran a considérablement réduit les taux de mortalité maternelle. Cependant, le schéma des causes de mortalité qui est. resté stable pendant plusieurs années est. toujours préoccupant. Le but de cette étude est. d’expliquer les expériences des agents iraniens de surveillance de la mortalité maternelle et de proposer des stratégies pour améliorer ce système.

**Méthodes:**

Cette recherche est. une étude qualitative et a été effectuée pour développer des stratégies qui se feront en deux phases. Dans la première phase, un échantillonnage ciblé est. effectué et les données sont utilisées par le biais d’entretiens semi-structurés basés sur les expériences des agents iraniens du système de surveillance de la mortalité maternelle au ministère de la Santé et dans certaines universités iraniennes (à Shiraz, Isfahan, Téhéran, Zahedan, Alborz, Shahrekord) seront collectées. De plus, au cours de cette phase, certaines parties des données seront collectées grâce à la participation aléatoire de chercheurs aux comités de mortalité maternelle dans certaines universités. Afin de réaliser la deuxième phase, selon les résultats ci-dessus, un groupe d’experts est. considéré pour discuter des meilleures stratégies pour améliorer la surveillance de la mortalité des mères iraniennes.

**Discussion:**

L’analyse des systèmes de surveillance de la mortalité maternelle nécessite d’évaluer les expériences des agents qui sont les décideurs de ce système et peuvent être efficaces pour identifier ses défis. Cette analyse et le développement de stratégies ultérieures peuvent garantir que les indicateurs de santé maternelle restent dans la portée des normes internationales ou même au-delà de ces normes dans les universités de l’Iran et des pays avec des systèmes de surveillance similaires.

## Plain English summary

The maternal mortality surveillance system, which includes processes such as the registration of maternal deaths, data collection, formation of death committees, and subsequently, design of the death preventive interventions, has been operating in Iran and other countries for many years. Although the implementation of this system has been successful in reducing death rates, the pattern of death causes in the form of bleeding and gestational hypertension has remained the same for years. As such, stop in the downward trend of the deaths can be predicted. Accordingly, the present study aims to analyze maternal mortality surveillance system in order to formulate strategies for the improvement of this system after analysis. Strategies can provide policymakers with the highest insight so that they can provide a rational and defensible basis for future decision-making and orientations on the pregnant women health index.

In the first phase of the research, the processes of this system will be analyzed by interviewing the maternal mortality surveillance system actors in the Ministry of Health and Medical Education and the selected universities of Iran and the shortcomings will be identified. In the second phase, based on the obtained analysis, a panel of experts is formed to develop strategies for the improvement of the maternal mortality surveillance system. This study seems to provide comprehensive strategies for the betterment of the maternal mortality surveillance system in order to improve the status of maternal health index and eliminates all causes of mortalities.

## Background

Identification and notification of death, its review, and the presentation of the intervention strategies are among the most valuable programs in countries to achieve a reduction in maternal death [1]. In this regard, the WHO, along with other participating organizations, has provided guidelines for monitoring maternal mortality and identifying the avoidable factors contributing to these deaths [2]; this program has been introduced in Iran as the Iranian Maternal Mortality Surveillance System. In this system, which began to work since 2000, the surveillance cycle begins with the death of the mother and, then, the inquiry team collects the death-related data. After examining the questionnaires by the inquiry team, the Maternal Mortality Committee analyzes the avoidable causes and, then, designs appropriate interventions and puts under surveillance the implementation of them based on this analysis [3, 4].

Through this system, the Islamic Republic of Iran has undertaken long-term and short-term planning on maternal health and in recent years has been identified by the WHO as one of the triumphant countries in achieving the fifth Millennium Development Goal (A development from 51 deaths per 100,000 in 2000 to 16 deaths per 100,000 in 2017) [5]. Although this is considered to be a remarkable success, it will be very difficult to maintain and improve these indicators since, according to the Sustainable Development Goals, this figure should be reduced to the ratio of 9 deaths per 100,000 by 2030. Accordingly, there is a need for more effort and interventions beyond existing programs [3, 6].

Currently, one of the most important goals of the Ministry of Health and Medical Education to maintain and improve this index is to prevent the recurrence of repetitious deaths, that is, the repeatable accidents under the same circumstances that, despite the constant efforts of the experts, have not been successfully achieved [7]; so that, bleeding and hypertension have been for many years the leading causes of maternal deaths throughout the country, even in the more benefited provinces [8]. Thus, although the maternal mortality rate is acceptable and even less than the world average, the causes of deaths are still worrisome that is due to the fact that they are highly preventable and, thus, their prevalence is unacceptable [9].

Unfortunately, mortalities with the mentioned causes occur in all provinces of Iran continually and with almost similar causes and conditions. After each death a compulsory and out-of-duty investigation begins and then ends immediately, and after sometimes another death occurs with the same panel and this defective cycle is repeated time and again. As such, a careful examination of all these factors seems to be essential [3]. This type of study attempts to interview the actors involved with the mortality surveillance system in order to analyze and identify problems related to this system in the context of the aforementioned processes and the contents related to the implementation of these processes. Then, based on the findings, strategic, comprehensive, and helpful interventions will be done in line with the enhancement of the maternal mortality surveillance system in order to improve maternal health index and eliminate all death-related causes. These strategies, which are defined as a systematic and organized effort for making decisions and taking fundamental actions, and are designed with the help of stakeholders [10], can be considered as a helpful method for the Ministry in dealing with the changes in the conditions of maternal health system and, consequently, reproductive status of women. Our assumption is that these strategies, functioning as an interventional model, examine all the causes related to the quality deficiency of the maternal mortality surveillance system. Given the impact of different, social, cultural, and health factors on the maternal health system, these strategies can affect the quality of the health-related services. Accordingly, this study aims to explain the experiences of the maternal mortality surveillance system actors and present strategies for the improvement of this system.

## Methods

This research is a qualitative study to develop strategies, approved by the Ethics Committee of Tarbiat Modares University, Tehran, Iran (IR. MODARES. Rec 1397.267) and study will be conducted in the fields of health and treatment of the Ministry of Health and the selected Iranian national universities. The selected universities in this study include three type I universities (Shiraz, Isfahan, Tehran), one type II university (Zahedan), and two type III universities (Alborz, Shahrekord). Tehran University of Medical Sciences was randomly selected from three Tehran Universities of Medical Sciences (Shahid Beheshti, Tehran and Iran). It was selected because we need to have one of the capital’s medical sciences universities in the research process. Alborz University of Medical Sciences is selected because of the immense cultural diversity in this province that is due to the over-migration. Accordingly, different implementation of the maternal mortality surveillance system is more possible than other provinces. Shiraz University of Medical Sciences is considered for being a pioneer in the development and implementation of some maternal health related programs (Accreditation Programs). Isfahan University of Medical Sciences is selected because maternal mortality rate in this university shows a decreasing trend during the last 3 years. Zahedan University of Medical Sciences is selected among the other medical sciences universities of the country because of the high maternal mortality rate of this university. Finally, Shahrekord University of Medical Sciences is selected among other medical sciences universities of the country because of the low maternal mortality rate of this university. At all stages of the study, informed written and oral consent will be obtained of all participants. This research will be conducted in two phases. In the first phase, the processes of maternal mortality surveillance system are analyzed qualitatively and through using a directed approach. In fact, using this analysis, the experiences of the surveillance system actors about the challenges of this system will be discovered and, after being completed, the codes base on the available categories, subcategories and final or main categories will be identified. In order to conduct the second phase of the research, a panel of experts is set up with the aim of considering the best strategies for the improvement of the Iranian maternal mortality surveillance system by considering the above analysis. In order to formulate these strategies, several meetings will be organized with the participation of the Ministry’s maternal health actors, stakeholders, and researchers of the study. In these sessions, the assessment of the internal and external environments in relation to the implementation of the Iranian maternal mortality surveillance system will be done by considering the obtained analysis, then, balancing these points, the strategies will be formulated.

### Study design

#### First phase: qualitative study (explaining the actors’ experiences)

The first phase of the study is designed to answer this question that “How are the actor’s experiences for Iranian maternal mortality surveillance system?” This study will be conducted using a qualitative content analysis method.

##### Sampling method

Purposive sampling method will be used in this research, whereby the actors, through active participation in the research, will help the researcher in better understanding of the problem. Sampling will continue until data saturation is reached and the categories are repeated.

##### Participants

The research participants are the maternal health actors in the Ministry of Health and Medical Education and the selected universities of the country. Maternal health actors in the Ministry of Health and Medical Education: In the area of health, these actors include the head of the Maternal Health Department, the expert in maternal health and the head of family, population and school’s health department. In the field of treatment, they include the head of the midwifery department, the expert of mothers in the midwifery department, the head of the treatment and evaluation department, the head of hospitals affairs, the head of the clinical excellence department, deputy of treatment and director of treatment.

Maternal health actors in the selected universities: In the field of the Health Deputy, these actors include the head of the maternal health department, the expert in maternal health and the head of family, population and school’s health department. In the field of the treatment deputy, these include the head of the midwifery department, the expert of mothers in the midwifery department, the head of the treatment and evaluation department, the head of hospitals affairs, the head of the clinical excellence department, deputy of treatment and director of treatment. In the field of educational deputy, they include educational assistant, the gynecologist member of the mothers’ mortality and morbidity committee, the head of gynecology department and the head of midwifery department of medical as well as nursing and midwifery schools and the heads of these faculties. In the referral hospitals they include the hospital head, the hospital manager, maternity officer, the head of mortality and morbidity committees, matrons and educational supervisors.

##### Research environment

The health deputy of the Ministry of Health and Medical Education, as well as the health and treatment deputy of the selected national universities (Alborz, Tehran, Isfahan, Zahedan, Shahrekord and Shiraz Universities of Medical Sciences) construct the research environment.

##### Inclusion and exclusion criteria

Inclusion criteria consists of having at least 2 years of working experience in the position of maternal health expert and being an active member of the university’s mortality and morbidity committees. For the hospital authorities, 1 year of experience in the relevant position is sufficient. The exclusion criteria will be the unwillingness of the participants at any phase of the research to continue their collaboration.

##### Data collection method

Data collection in this phase will be done through in-depth semi-structured interviews, field notes and observations. Interviews will be conducted with the maternal health actors at the Ministry Health and Medical Education and the selected universities, and field notes are based on their nonverbal behavior. In the interviews, the processes of implementing the Iranian maternal mortality surveillance system (Collecting maternal death data, Maternal death review, Intervention design and Execution of interventions) will be examined to determine its distance from the standards; moreover, the challenges and defects of implementing such a system will be evaluated. Observation is also done by the random participation of the researcher in one or more death committees of the selected universities in order to record how the maternal mortality surveillance system is implemented.

##### Data analysis method

Directed content analysis will be used for data analysis and interpretation. All 3 stages of preparation (convert interview to text), organize (the emergence of new categories by forming an unconstrained matrix) and reporting, will be considered [11].

##### Accuracy and reliability of the data

To assure the trustworthiness of the findings, the four criteria of credibility, dependability, confirmability, and transferability will be employed.

#### Phase II: strategies design

The second phase of the study is designed to answer this question that “what strategies are effective for Iranian maternal mortality surveillance system?” In order to conduct the second phase, sessions will be held considering the mentioned analysis and using focus group discussion method to find the best strategies for improving the Iranian Maternal Mortality Surveillance System.

##### Participants

The research population in this phase includes the head of the midwifery and reproductive health board, the head of women’s board, the head and expert of the Department of Maternal Health in the Ministry, the head of the Department of Midwifery, one faculty member of the Obstetrics and Gynecology Department, one faculty member of the Midwifery Department, stakeholders out of the health system (representative of the Medical Council, representative of the Forensic Medicine Organization, representative of the Broadcasting Organization, representative of the Welfare Organization, and representatives of the Provincial Government, Governor and Municipality).

##### Research environment

The sessions for the development of strategies will be held at the Ministry of Health and Medical Education.

##### Inclusion and exclusion criteria

Inclusion criterion is that the participants are required to have at least 2 years of relevant work experience. The exclusion criteria will be the unwillingness of the participants at any stage of the study sessions to continue their collaboration.

##### Data collection method

Data collection process in this phase is performed through focused group discussion, so that the views, judgments, and attitudes of the visiting professionals about the strategies used for the improvement of the surveillance system are considered in several sessions. It should be noted that before the sessions, the results of the first part of the research as well as the comparisons of maternal mortality surveillance systems in the world, are prepared as a protocol and are given to the stakeholders of the research by the researcher. The first session will be held with the participation of the representatives of the Health Ministry, the head of the department of the Midwifery of the Health Ministry, and the head of the department of Maternal Health at the Health Ministry. After presenting the general issues of the national system of maternal mortality surveillance and its significance in the field of health, the results of the interviews about the analysis of this system will be discussed. Given the mission of the Ministry in improving the national system of maternal mortality surveillance to reduce maternal deaths, and based on the obtained analysis, the context is provided for determining and selecting a number of stakeholders in order for the sessions to be held purposefully. In the next sessions, with the presence of the aforementioned members and selected stakeholders, the internal (strengths and weaknesses) and external (opportunities and threats) factors of the maternal mortality surveillance system will be assessed.

##### Data analysis

Data analysis is performed in other sessions through creating a strategic balance between the strengths, weaknesses, opportunities, and threats (SWOT). The formulation of the strategies is done through this balance. The matrix tables (decision matrix, prioritization and selection matrix, and final matrix) are then drawn to consider the most important strategies.

## Discussion

Many studies have emphasized the necessity of following maternal mortality surveillance system to reduce maternal deaths. According to these studies, decrease in the maternal mortality ratio during the recent years, compared to 1990s, has been related to the use of this system [12–16]. Moreover, examining the viewpoint of health workers about the maternal mortality system, other studies have had a positive attitude towards this system, and considered its flexibility, acceptability and sensitivity to be valuable [17, 18]. However, other studies have referred to underreporting of maternal deaths [1], failure to obtain the real causes of deaths, and the effect of health barriers on the system [7, 19, 20]. These studies have emphasized the need to modify surveillance system processes based on resolving structural problems and sensitizing health practitioners so that this system can be used as optimally as possible.

However, the evaluation of the processes of maternal mortality surveillance system by its actors is necessary so that we can formulate the essential strategies for improving the surveillance system based on the data obtained from the evaluation. In previous studies conducted in Iran, strategies developed in line with the maternal mortality surveillance system suggested that these strategies could be formulated solely with respect to deficiencies obtained from the report of the deaths or based on their causes. As such, none of these studies have had a complete and in-depth interview with the actors of this system to analyze it and use the results for the development of the strategies [9, 10].

The present study in the form of maternal mortality surveillance system analysis is expected that all major universities in Iran develop a coherent planning based on these strategies in order to solve major issues and problems in this regard. Additionally, as the maternal death system in all countries is based on the recommendations of WHO, FIGO, ICM, UNFPA and CDC, and all countries are required to follow this system in order to achieve a reduction in maternal deaths [21], the present study may also be appropriate for other countries.

By and large, these strategies can improve the organizational performance of the Ministry of Health and Medical Education and the related universities with regard to maternal health in Iran and other countries of the world, and deal appropriately with the rapidly changing situations in order to maintain the level of the maternal mortality standards at the level of international standards or even beyond them.

## Data Availability

Not applicable.

## Abbreviations

MMR: Maternal Mortality Ratio
WHO: World Health Organization
FIGO: International Federation of Gynecology and Obstetrics
ICM: International Confederation of Midwives
UNFPA: United Nation Population Fund
CDC: Center for Disease Control
MCMC: Medical Care Monitoring Center
IMMSS: Iranian Maternal Mortality Surveillance System
